# The space of Retzius: anatomy, imaging features, and patterns of disease spread

**DOI:** 10.1186/s13244-026-02403-4

**Published:** 2026-09-25

**Authors:** Sevtap Arslan, Deniz Berkay Sonu, Ali Enes Aldur, Musturay Karcaaltincaba, Deniz Akata, Mustafa Nasuh Ozmen, Ali Devrim Karaosmanoglu

**Affiliations:** 1 Department of Radiology, Hacettepe University School of Medicine, Ankara, Turkey; 2 Department of Anatomy, Hacettepe University School of Medicine, Ankara, Turkey

**Keywords:** Retropubic space, Urinary bladder, Urachus, Neoplasms, Hematoma

## Abstract

**Abstract:**

The space of Retzius is a clinically important but often overlooked extraperitoneal compartment that serves as a common site of disease involvement and a key pathway for the spread of pelvic and retroperitoneal pathology. Its complex anatomical relationship with other extraperitoneal/retroperitoneal spaces renders it prone to involvement in different pathologic processes. Most cases represent secondary involvement rather than primary pathology. CT and MRI play a pivotal role in depicting the anatomy of the space of Retzius, characterizing lesions, and identifying routes of disease extension. This review provides a comprehensive imaging-based overview of the anatomy of the space of Retzius and illustrates the imaging findings of a broad spectrum of diseases involving this region. We highlighted the anatomical pathways of disease spread, characteristic imaging features, and surgical considerations relevant to radiologists. Familiarity with these imaging patterns and anatomical relationships can improve diagnostic confidence, facilitate identification of the underlying disease process, and guide appropriate clinical management.

**Key Points:**

- **Question** Accurate recognition of diseases involving the space of Retzius is challenging because of its complex anatomy and diverse patterns of disease spread.
- **Findings** Most pathologies involving the space of Retzius represent secondary extension from adjacent compartments rather than primary disease.
- **Critical relevance statement** By integrating imaging anatomy, patterns of disease spread, and surgical relevance, this review provides a practical framework for evaluating abnormalities involving the space of Retzius, improves diagnostic confidence, facilitates identification of the disease process, and guides clinical and surgical management.

**Graphical Abstract:**

**Figure:**
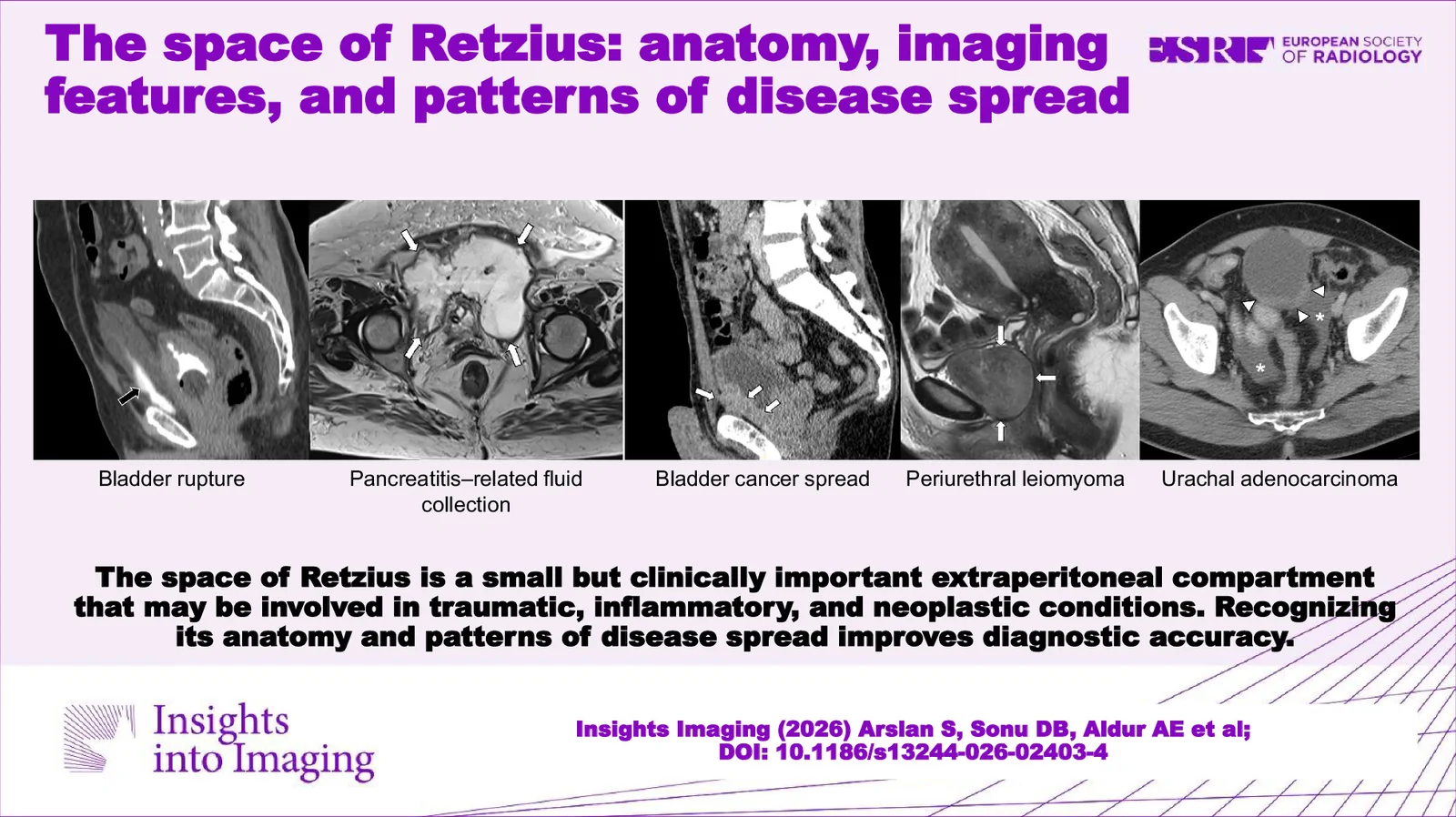

## Introduction

Anders Retzius, a Swedish anatomy professor, first described the extraperitoneal prevesical space as “cavum Retzii” or “retropubic space of Retzius”, which was later defined as “spatium retropubicum” in Latin [1]. With the advancements in different imaging technologies, it is now possible to visualize and evaluate the space of Retzius in greater detail than ever before. Initial assessment may be performed using plain radiography or transabdominal ultrasound; however, the pubic symphysis is a major restrictive barrier, causing X-ray attenuation and acting as an acoustic reflective interface. In daily practice, cross-sectional imaging modalities, computed tomography (CT) and magnetic resonance imaging (MRI), are more commonly utilized, as they allow a comprehensive evaluation of this area. Multiplanar imaging and reformatted views further improve the depiction and characterization of disease processes.

Our major goal in this review is to provide a comprehensive imaging-based overview of the anatomy as well as the diseases affecting the space of Retzius.

## Anatomy

The space of Retzius is a small extraperitoneal space mostly containing fat, loose connective tissue, and prostatic venous plexus (Santorini’s plexus). Median umbilical ligament, remnant of the embryological urachus, extending from the apex of the urinary bladder to the umbilicus, is also located in this area. The shape of this space is variable depending on the patient’s body habitus without a defined volume [2].

The nomenclature of this anatomical compartment is not entirely uniform. While some anatomical texts define the space of Retzius as the retropubic (inferior) portion of the broader prevesical space, others use the terms space of Retzius and prevesical space interchangeably. In this review, we adopt the latter terminology. The space of Retzius extends superiorly toward the umbilicus between the paired medial umbilical ligaments, which represent the distal obliterated segments of the umbilical arteries. From its superior to inferior extent, the space is bounded anteriorly by the transversalis fascia and the pubic symphysis. Posteriorly, it is delineated by the parietal peritoneum and the urinary bladder, while inferior boundaries are formed by the puboprostatic ligaments (males) or pubovesical ligaments (females), and the superior fascia of the levator ani muscle. The pubic rami and obturator internus muscles define the lateral margins of the space (Supplementary Materials 1 and 2). The umbilicovesical fascia extends from the umbilicus to the bladder, enveloping the urachus, obliterated umbilical arteries and urinary bladder, and forms the boundary between the space of Retzius and the paravesical space [2–4].

A cadaveric anatomical study involving the injection of contrast material into various extraperitoneal compartments demonstrated that the space of Retzius is in continuity with the paravesical and posterior pararenal spaces in all subjects. Continuity with the perirenal, anterior pararenal, and perirectal spaces was also observed in 75%, 50%, and 25% of cases, respectively [5]. Communications with the rectus sheath, inguinal canal, and femoral sheath have also been demonstrated in a different cadaveric study [6]. These intricate anatomical connections predispose the space of Retzius to involvement by several disease processes originating from different anatomical compartments.

## Diseases involving the space of Retzius

### Bladder rupture

Urinary bladder trauma commonly occurs in the setting of high-energy trauma and is frequently associated with pelvic fractures. Rarely, bladder injury may arise as an iatrogenic complication during surgical procedures involving the bladder or adjacent pelvic organs. Clinical presentation may include hematuria, difficulty with passing urine, and lower abdominal pain [7]. However, clinical diagnosis is often challenging due to the nonspecific symptoms and presence of other concomitant injuries related to major trauma. Therefore, imaging plays a fundamental role in diagnosis.

Bladder injuries are traditionally classified into five types: contusion, intraperitoneal rupture, interstitial bladder injury, extraperitoneal rupture (simple or complex), and combined rupture. The primary diagnostic modalities for bladder injury include CT cystography or conventional cystography, cystoscopy, excretory-phase CT imaging, or intravenous pyelography [8, 9].

**Fig. 1:**
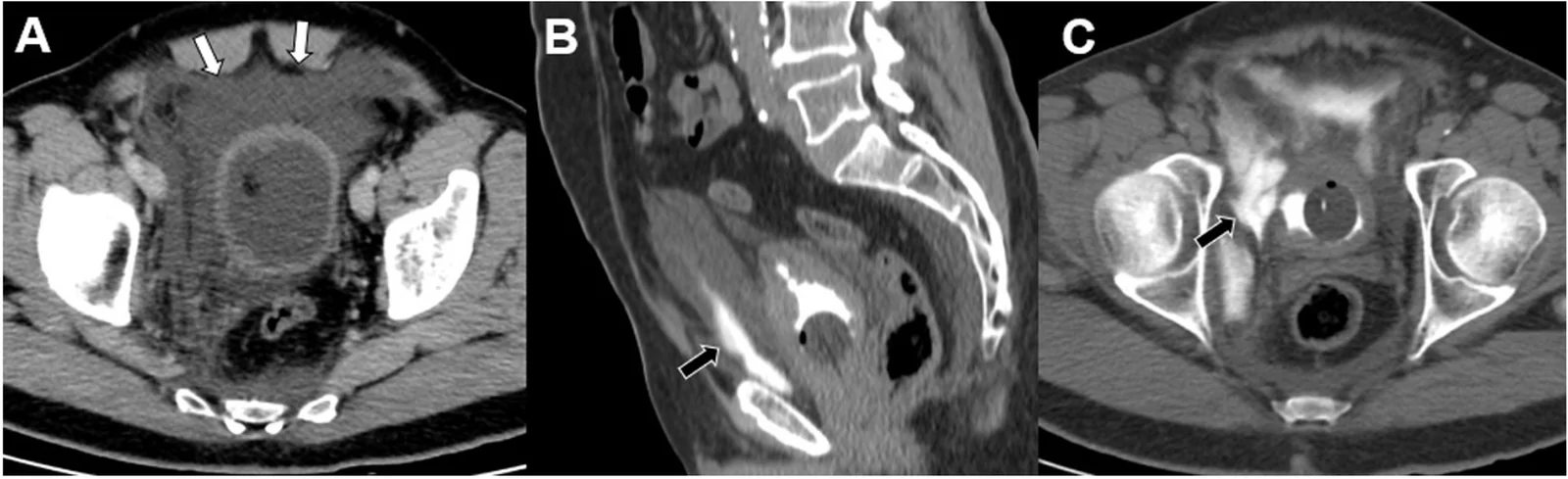
Extraperitoneal bladder rupture into the space of Retzius in a 75-year-old male patient after transurethral resection of the prostate. Axial plane contrast-enhanced CT image (**A**) shows fluid collection in the space of Retzius (white arrows) and along the right paravesical space. Sagittal (**B**) and axial (**C**) plane excretory-phase CT images reveal contrast media extralumination (black arrows) from the bladder into the space of Retzius

Extraperitoneal rupture represents the most frequent injury pattern, accounting for 80–90% of the cases, and is commonly associated with pelvic fractures or penetrating trauma. The area of injury is typically located in the anterolateral aspect of the bladder base. On CT cystography or excretory-phase CT, extraperitoneal bladder rupture is characterized by extravasation of contrast material into extraperitoneal spaces surrounding the bladder, including the space of Retzius and the paravesical spaces (Fig. 1). In simple extraperitoneal rupture, contrast initially accumulates in the space of Retzius and may extend into the bilateral paravesical spaces, producing the characteristic “pelvic molar tooth” sign. In contrast, complex extraperitoneal rupture demonstrates extension of contrast beyond the perivesical region, with dissection along multiple fascial planes and into adjacent extraperitoneal spaces. A detailed knowledge of abdominopelvic anatomy and fascial pathways is therefore essential to prevent misinterpretation of complex extraperitoneal bladder rupture. Most bladder injuries can be managed conservatively with a urinary catheter [8–11].

### Urachal anomalies

**Fig. 2:**
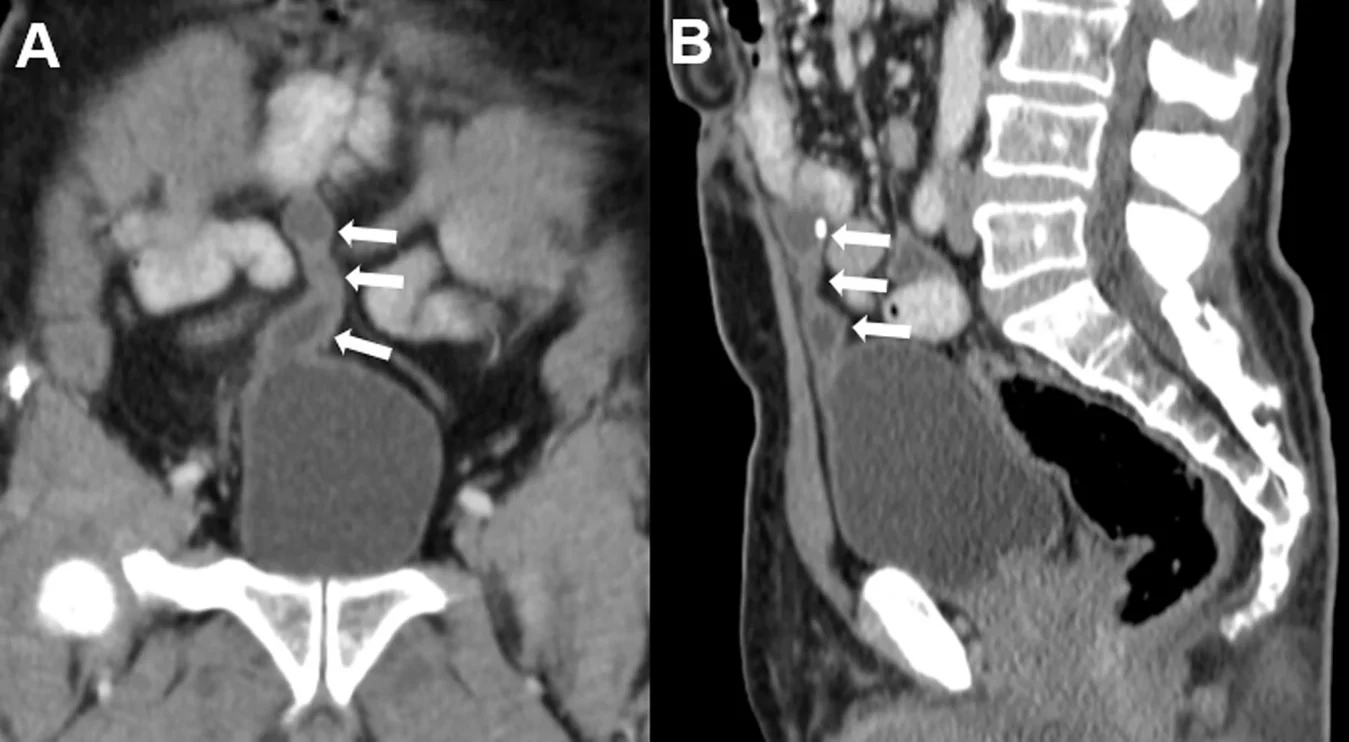
Coronal (**A**) and sagittal (**B**) CT images demonstrate a tubular-shaped lesion (arrows) extending from the bladder apex to the umbilicus, consistent with a vesicourachal diverticulum, in a 73-year-old male patient with a history of gastric cancer

**Fig. 3:**
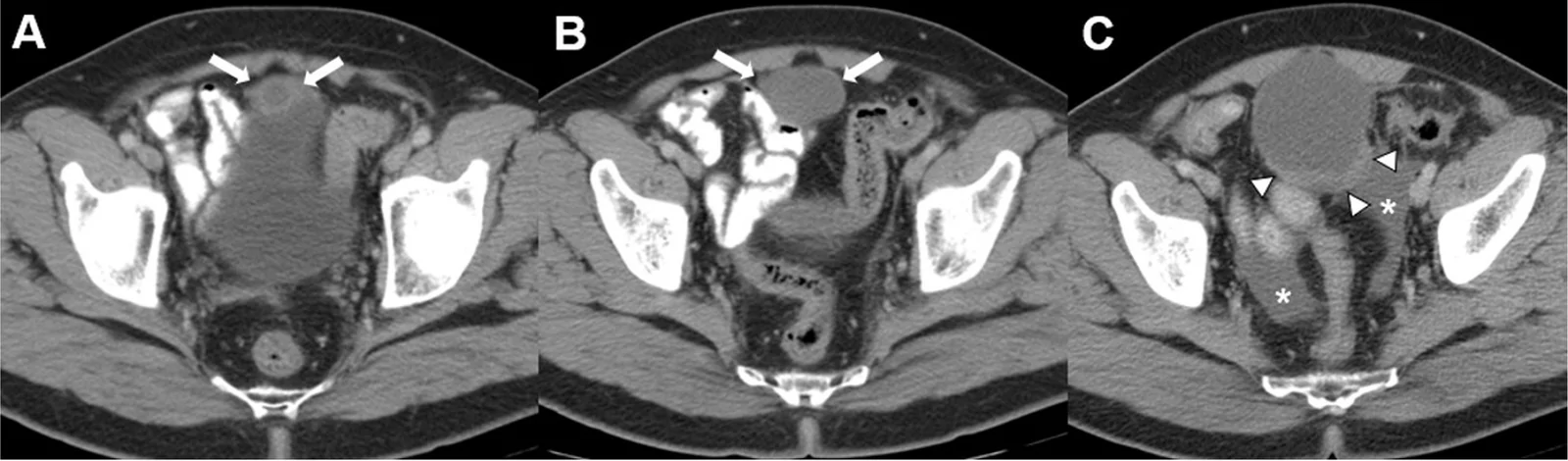
Consecutive axial CT images (**A**, **B**) demonstrate a cystic lesion (arrows) adjacent to the bladder apex, consistent with a urachal cyst (arrows). Follow-up axial CT image obtained 2 years later (**C**) shows interval enlargement of the lesion with irregular wall thickening (arrowheads) and associated pelvic free fluid (asterisks), raising suspicion for malignant transformation. Postsurgical histopathological examination confirmed mucinous adenocarcinoma arising from the urachal cyst

Urachus is an embryonic structure that extends from the bladder dome to the umbilicus and connects the fetal urinary bladder to the allantois. Obliteration of the urachus usually begins during fetal development, around the 12th week of gestation, although completion may extend into the postnatal period, resulting in the formation of the median umbilical ligament. Failure of this obliteration results in urachal anomalies, with an estimated incidence of approximately 1 in 5000 individuals. Urachal anomalies are frequently detected incidentally on imaging performed for other reasons. When symptomatic, they may present with lower abdominal pain, umbilical discharge, recurrent infections, hematuria, or as a palpable midline mass. Although the reported prevalence of urachal anomalies varies among published series, four major types have been consistently described: patent urachus (10–48%), urachal cyst (31–43%), umbilical–urachal sinus (18–43%), and vesicourachal diverticulum (3–5%) [12–15]. Urachal anomalies may present as cystic or tubular lesions in the space of Retzius. Patent urachus appears as a continuous tubular tract between the bladder and umbilicus. Urachal cysts present as midline fluid collections, most commonly near the bladder dome. An umbilical–urachal sinus is observed as a blind-ending dilatation at the umbilical end, whereas a vesicourachal diverticulum is a focal protrusion from the bladder apex (Fig. 2, Supplementary Materials 3 and 4). Urachal malformations may get secondarily infected and manifest as inflammatory masses or abscesses on imaging. Rarely, these remnants may undergo malignant transformation, which gives rise to urachal carcinoma (Fig. 3) [12, 13].

### Neoplastic and non-neoplastic lesions

**Fig. 4:**
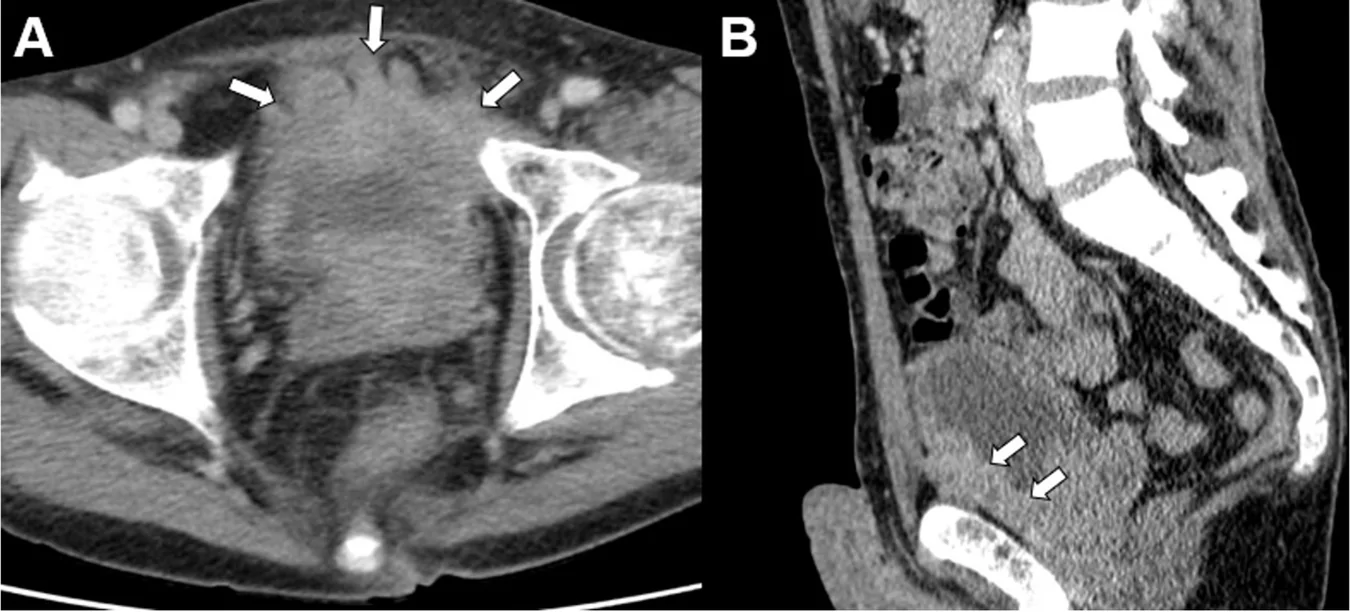
Secondary involvement of the space of Retzius by bladder cancer. Contrast-enhanced axial (**A**) and sagittal (**B**) CT images demonstrate tumoral invasion into the space of Retzius (arrows)

Neoplastic involvement of the space of Retzius is relatively uncommon but may occur through hematogenous metastasis, direct tumor invasion from adjacent pelvic structures, or tumor implantation following surgical intervention. Local recurrence within this space may be observed after resection of urogenital malignancies such as bladder, prostate, or gynecologic cancers (Fig. 4, Supplementary Materials 5–8).

**Fig. 5:**
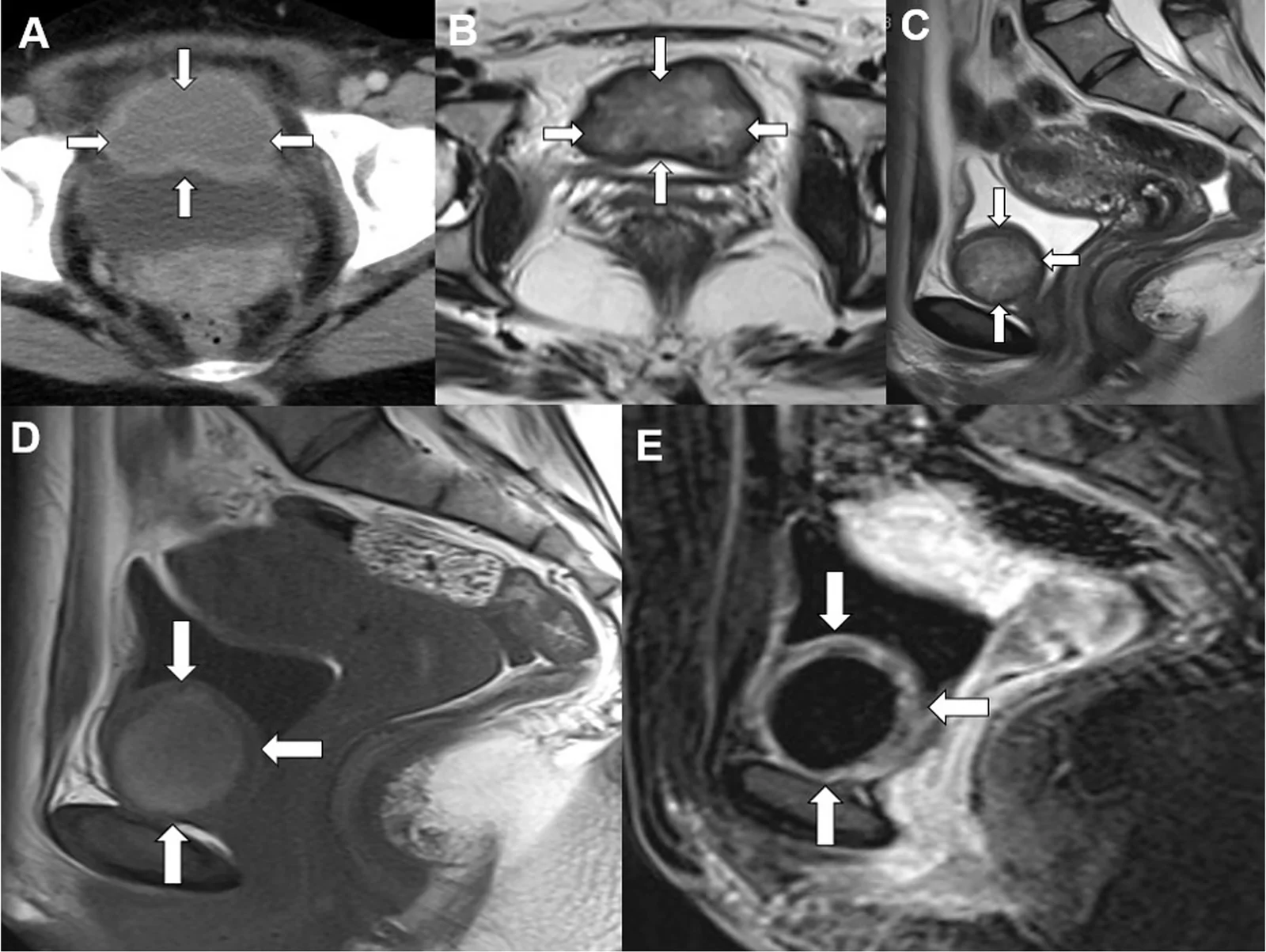
Endometrioma in the space of Retzius. A 33-year-old woman with a history of a caesarean section 6 months earlier presented with dysuria. A non-contrast CT image (**A**) demonstrates a hyperdense, lobulated mass in the space of Retzius. Axial (**B**) and sagittal (**C**) T2-weighted, sagittal T1-weighted (**D**), and sagittal post-contrast subtraction (**E**) MR images show a hemorrhagic mass without internal enhancement. The imaging findings were suggestive of endometrioma, and the diagnosis was confirmed by histopathologic examination following surgical resection

Benign non-neoplastic lesions, including endometriosis, may also develop within the space of Retzius (Fig. 5) [4]. Endometriotic implants typically present as ill-defined soft-tissue nodules or fibrotic masses, often demonstrating variable T1 hyperintensity related to hemorrhagic components.

**Fig. 6:**
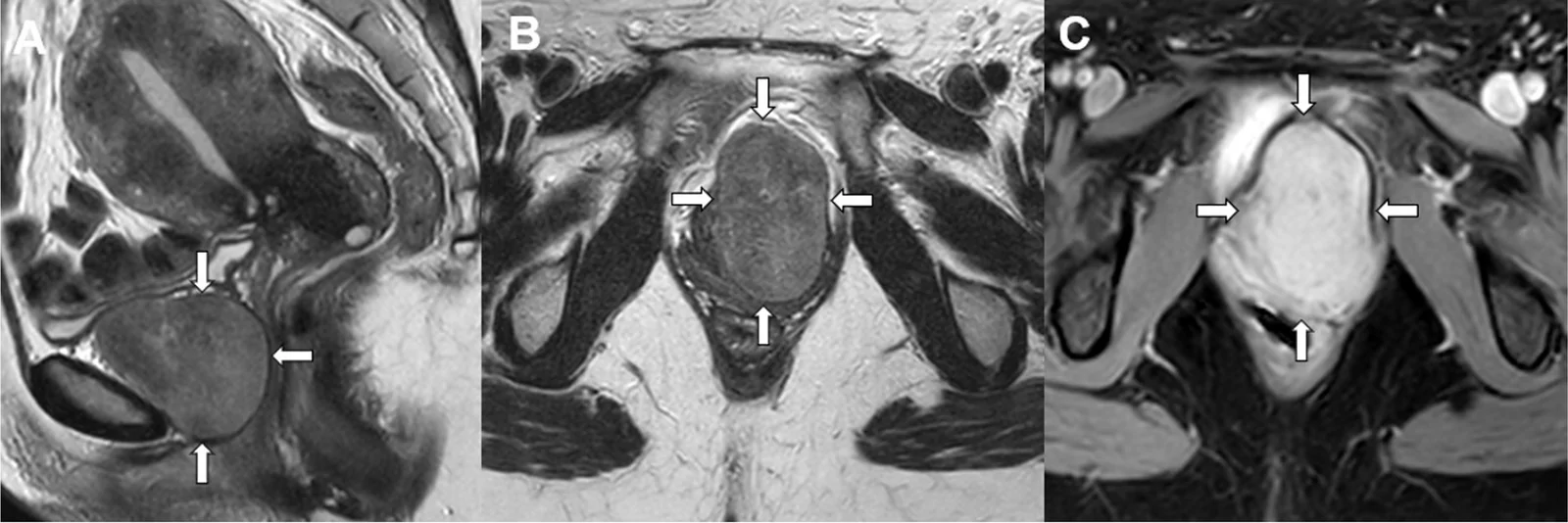
Periurethral leiomyoma in the space of Retzius. Sagittal and axial T2-weighted (**A**, **B**) and axial post-contrast T1-weighted (**C**) MR images demonstrate a T2-hypointense, homogeneously enhancing round mass in the space of Retzius (arrows)

Primary tumors affecting the space of Retzius are rare. Benign neoplasms such as leiomyoma and venolymphatic malformations, as well as uncommon malignant tumors including immature teratoma, ependymoma, and malignant glomus tumor, may be observed in this location. Retroperitoneum is an uncommon location for deep soft-tissue leiomyomas [16–18]. Retroperitoneal leiomyomas usually present as well-circumscribed solid masses with iso- to low signal intensity on T1-weighted images and low to intermediate signal intensity on T2-weighted images (Fig. 6). Post-contrast enhancement patterns vary and often resemble uterine fibroids. Some lesions may contain myxoid stroma or cystic degeneration, resulting in internal T2 hyperintensity. Given these imaging features, differentiation of leiomyoma from other retroperitoneal tumors based solely on imaging is often challenging [19–21]. Disseminated peritoneal leiomyomatosis is a rare condition characterized by multiple smooth-muscle nodules developing along the submesothelial tissues of the peritoneum and may occasionally involve the space of Retzius (Supplementary Material 9).

Venolymphatic malformations typically manifest as multiloculated cystic lesions with fluid–fluid levels and demonstrate minimal to gradual peripheral enhancement [22, 23]. When located in the pelvis, they may spread along extraperitoneal fascial planes and extend into the space of Retzius (Supplementary Material 10).

**Fig. 7:**
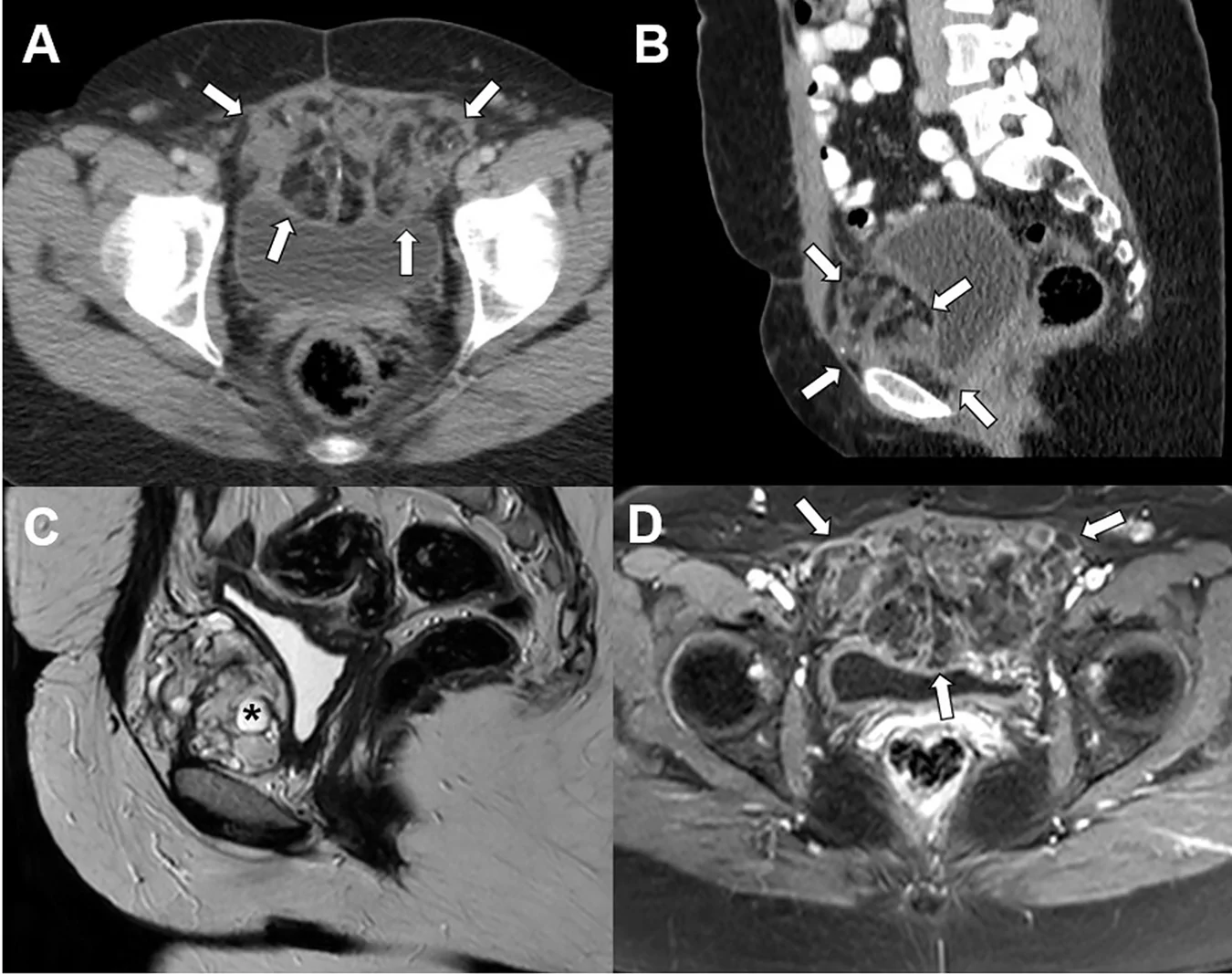
Pathologically proven malignant immature teratoma in the space of Retzius. Axial and sagittal (**A**, **B**) CT images show a lobulated macroscopic fat-containing mass in the space of Retzius (arrows). **C** Sagittal T2-weighted MR image demonstrates cystic areas within the mass (asterisk). **D** Axial post-contrast T1-weighted MR image shows heterogeneous enhancement of the lesion

Teratomas are germ cell tumors composed of tissues derived from the three germ layers and most commonly arise in the ovaries and testes. However, extragonadal teratomas have also been reported, with the sacrococcygeal region being the most common site [24]. On rare occasions, teratomas may involve the space of Retzius (Fig. 7). In contrast to mature cystic teratomas, immature teratomas typically present as a heterogeneous mass with small foci of fat and prominent solid components, which may contain calcifications [25].

**Fig. 8:**
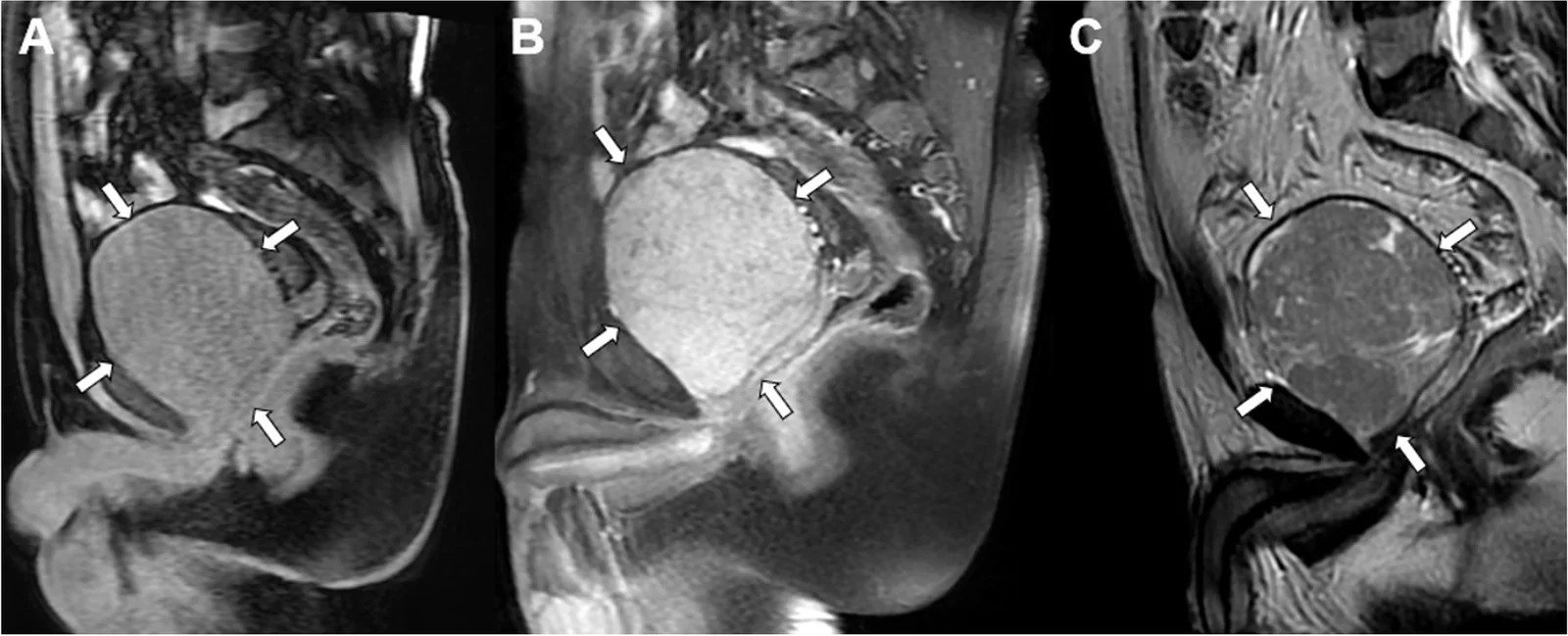
Histopathologically confirmed ependymoma in the space of Retzius. Sagittal pre- and post-contrast (**A**, **B**) T1-weighted and sagittal T2-weighted (**C**) MR images show a large, round mass in the space of Retzius (arrows), showing homogeneous enhancement (arrows)

Ependymomas are neuroepithelial tumors with ependymal differentiation that most frequently involve the ventricular system and appear as well-defined soft-tissue masses with heterogeneous enhancement and occasional cystic degeneration. Extraneural ependymomas are exceedingly rare but have been reported within the space of Retzius (Fig. 8) [26].

**Fig. 9:**
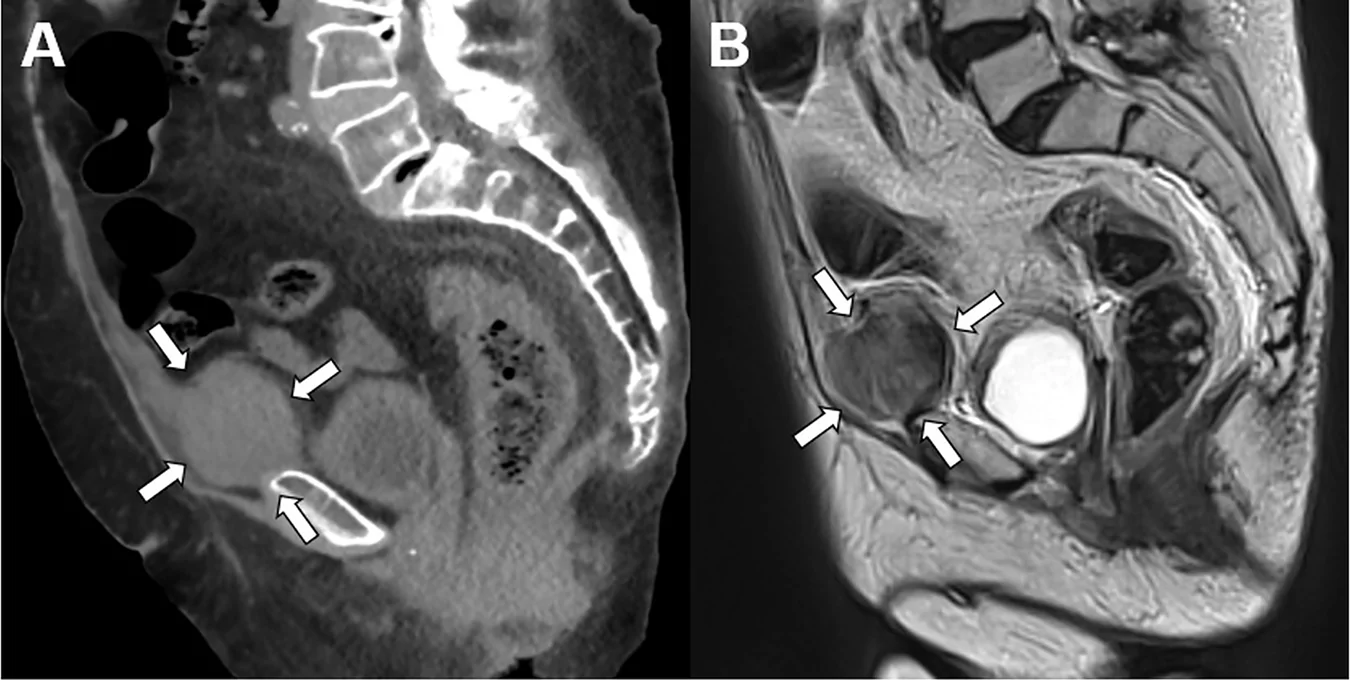
Biopsy-proven malignant glomus tumor of the space of Retzius in an 84-year-old woman. Sagittal unenhanced CT (**A**) and sagittal T2-weighted MR image (**B**) demonstrate a well-circumscribed, spherical mass in the space of Retzius (arrows). Intravenous contrast material was not administered in each examination due to impaired renal function

Glomus tumors are uncommon vascular neoplasms that classically arise in the subungual region. Malignant glomus tumors are extremely rare and tend to occur in deep soft tissues, often presenting as hypervascular solid masses with avid contrast enhancement and high T2 signal intensity. Visceral, peritoneal, and other abdominal locations of glomus tumors are exceedingly rare (Fig. 9) [27–30].

Rare tumors involving this space have been reported in the literature, mostly as anecdotal cases, including aggressive angiomyxoma, lipoma, solitary fibrous tumor, hemangiopericytoma, and xanthogranulomatous pseudotumor [21, 31–34].

### Fluid collection, hematoma, and fistula

Postoperative hematomas or fluid collections—including seromas, urinomas, lymphoceles, and infected collections—may involve the space of Retzius (Supplementary Material 11). Rarely, hematomas arising from the rectus sheath or femoral sheath may extend into this space [4]. Small amounts of hematoma or fluid collection are generally managed conservatively; however, surgical or percutaneous intervention may be required in cases where hematomas exert significant mass effect or cause hemodynamic instability.

**Fig. 10:**
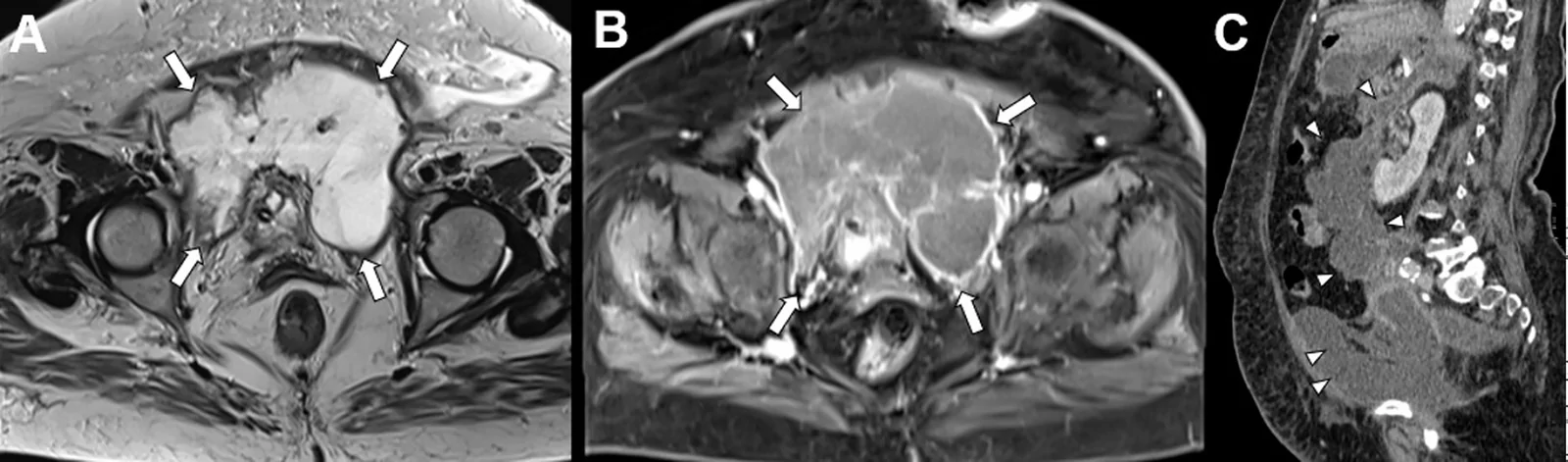
Necrotizing pancreatitis–related fluid collection extending into the space of Retzius. Axial T2-weighted (**A**) and post-contrast T1-weighted (**B**) MR images demonstrate a peripherally enhancing, multiloculated fluid collection in the space of Retzius extending into the paravesical spaces, producing the “molar tooth” sign (white arrows). A sagittal contrast-enhanced CT image (**C**) demonstrates continuity of the peripancreatic fluid collection into the retroperitoneum, as well as the space of Retzius (white arrowheads)

Although anatomically distant from the primary site, pancreatitis-related fluid collections may extend into the space of Retzius on rare occasions (Fig. 10) [4]. Retroperitoneal inflammatory fluid can track inferiorly along established interfascial planes—including the anterior pararenal space, paracolic gutters, and pelvic extraperitoneal compartments—and eventually reach this area. Such inferior extensions may mimic primary pelvic infection or malignancy. Awareness of this pathway and careful correlation with accompanying imaging findings are crucial for accurate diagnosis.

Following urogynecological procedures, fistulous tracts originating from the bladder or urethra may dissect into the space of Retzius. Imaging may demonstrate extraperitoneal contrast leakage on CT cystography, urinoma formation, or gas- or fluid-containing sinus tracts extending within the space of Retzius (Supplementary Material 12). Awareness of these complications is important as they may present with nonspecific pelvic pain or persistent postoperative infection. It can easily be overlooked without targeted imaging evaluation.

### Air in the Retzius space

Free air within the space of Retzius may be observed in the early postoperative period or in association with bowel perforation. In patients without recent surgical intervention, extraperitoneal gas should raise concern for perforation of a retroperitoneal hollow viscus.

**Fig. 11:**
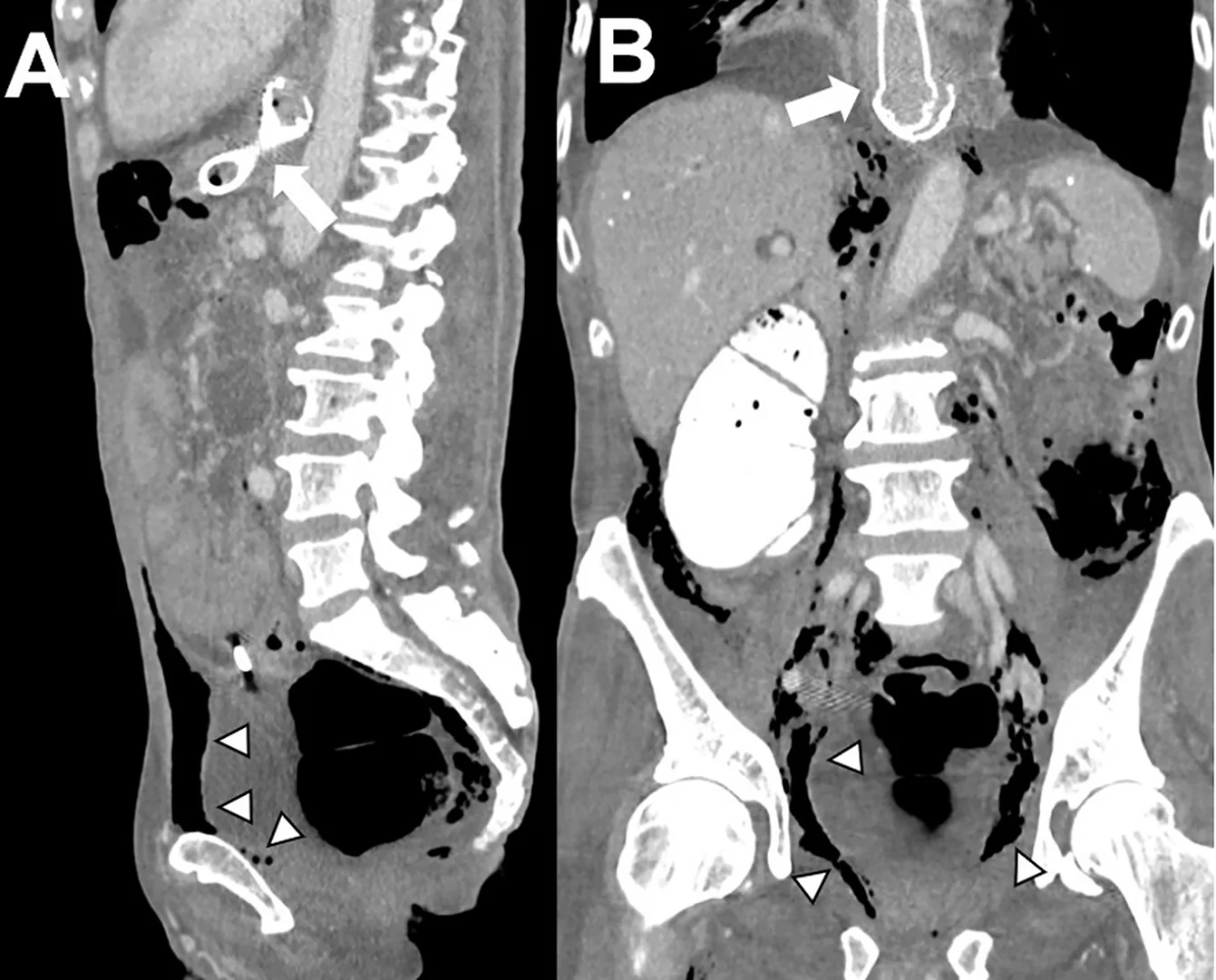
Sagittal and coronal reformatted CT images demonstrate free air in the space of Retzius and paravesical spaces (arrowheads in **A** and **B**) secondary to complicated esophagojejunal stenting (arrows). Note the air densities adjacent to the stent with inferior extension to the level of the space of Retzius

Perforation of several retroperitoneal gastrointestinal structures may result in retroperitoneal air leakage, which can descend toward the pelvis and accumulate in the space of Retzius through continuity between the extraperitoneal compartments (Fig. 11). On CT, air outlining the bladder, air dissection along the pelvic soft-tissue planes, or detection of localized air within the prevesical space are common. In these patients, free air may be very limited or completely absent in intraperitoneal compartments.

## Surgical considerations

Beyond its involvement in a wide spectrum of pathological processes, the space of Retzius represents an important surgical corridor for multiple pelvic procedures, such as radical prostatectomy, radical hysterectomy, pelvic lymphadenectomy, inguinal hernia repair, and reconstructive urogynecologic surgery. The principal surgical applications of this space are summarized in Table 1 [35–42]. The space of Retzius contains Santorini’s plexus, an important surgical landmark. Careful identification and control of this plexus are essential to minimize intraoperative blood loss during pelvic procedures involving the space of Retzius.

**Table 1:** Surgical applications of the space of Retzius

Specialty	Procedure(s)	Surgical relevance of the space of Retzius
General surgery	Transabdominal preperitoneal (TAPP) and extended totally extraperitoneal (eTEP) hernia repair	The space of Retzius provides the medial extraperitoneal surgical corridor in both eTEP and TAPP hernia repair, allowing exposure of the myopectineal orifice, identification of Cooper’s ligament, reduction of direct hernia sacs, and adequate medial mesh placement. Its continuity across the midline also facilitates bilateral inguinal hernia repair.
Urology	Radical prostatectomy	Preservation of the Retzius space maintains the anterior supporting structures of the bladder neck and urethra and has been associated with improved early urinary continence and postoperative sexual function.
Urology	Inflatable penile prosthesis implantation	The prosthesis reservoir is commonly positioned within the Retzius space.
Gynecology/urogynecology	Burch colposuspension and Marshall–Marchetti–Krantz urethropexy	Retropubic dissection provides access to the bladder neck, periurethral tissues, and Cooper’s ligament for restoration of urethral support in stress urinary incontinence.
Gynecology/urogynecology	Retropubic mid-urethral sling (TVT)	The sling traverses the space of Retzius.
Gynecologic oncology	Pelvic lymphadenectomy	The space of Retzius provides orientation to the anterior pelvic anatomy and facilitates entry into the paravesical spaces, particularly in patients with frozen pelvis.
Gynecologic oncology	Pelvic exenteration	The space of Retzius serves as the principal anterior dissection plane during radical pelvic resection.
Gynecologic oncology	Radical hysterectomy	The space of Retzius enables anterior bladder mobilization and facilitates separation of the bladder from the cervix and upper vagina during radical pelvic surgery.

The space of Retzius is routinely accessed in transabdominal preperitoneal repair for recurrent inguinal hernia and in suprapubic extended totally extraperitoneal repair for infraumbilical hernias. It provides a key medial corridor to expose the myopectineal orifice, allowing surgeons to clearly identify landmarks like Cooper’s ligament and perfectly position the mesh medially beneath the pubic bone [35, 36]. Following preperitoneal hernia repair, prosthetic mesh may be identified on CT as a thin linear or slightly hyperattenuating structure along the posterior aspect of the anterior abdominal wall, although its visibility depends on the mesh material. Expected postoperative findings include mild soft-tissue stranding, small amounts of preperitoneal air, and small seromas, whereas mesh-related complications include hematoma, infection, abscess formation, and mesh failure [37, 38].

Urogynecologic interventions also frequently require traversal of this compartment. In the retropubic mid-urethral sling procedure for stress urinary incontinence, a synthetic sling is placed retropubically beneath the urethra within the prevesical space (Supplementary Material 13) [39]. Burch colposuspension and Marshall–Marchetti–Krantz operations are retropubic urethropexy techniques for stress urinary incontinence in which periurethral tissues and the bladder neck are elevated within the retropubic space and anchored to the Cooper ligaments to re-establish urethral support (Supplementary Material 14) [40].

**Fig. 12:**
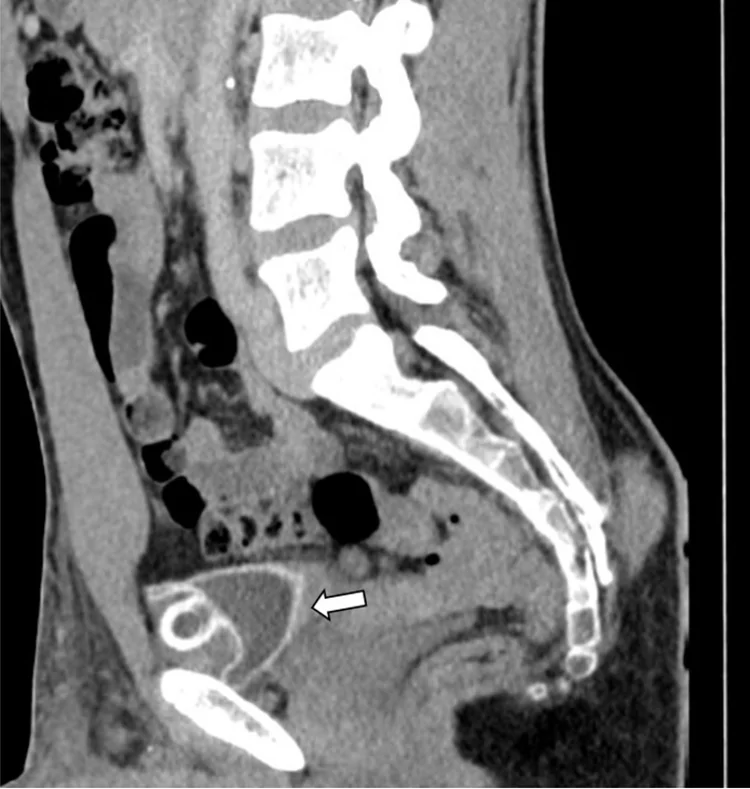
Sagittal reformatted CT image shows the reservoir of a three-piece penile prosthesis in the space of Retzius (arrow)

During inflatable penile prosthesis implantation, the reservoir is frequently positioned within the space of Retzius, highlighting the clinical relevance of this area in surgical planning (Fig. 12) [41]. Traditionally, radical prostatectomy has been performed through the space of Retzius, which provides direct access to the prostate and periurethral structures. The more recently introduced Retzius-sparing radical prostatectomy, which uses a posterior approach, preserves the neurovascular bundle and bladder neck support, resulting in earlier recovery of urinary continence and improved postoperative sexual function. Compared with the conventional anterior approach, Retzius-sparing surgery has been associated with a higher incidence of lymphocele formation and urinary retention, but a lower risk of de novo inguinal hernia and bleeding requiring blood transfusion. These benefits should, however, be balanced against the greater technical complexity of the procedure and the possibility of higher positive surgical margin rates in selected patients depending on tumor characteristics and surgeon experience [42–44].

## Conclusion

Despite its relatively small size, the space of Retzius is a clinically important extraperitoneal compartment that may be involved in a broad spectrum of traumatic, inflammatory, neoplastic, and postoperative conditions. As involvement of this space most often reflects secondary spread rather than primary disease, attention should be directed to other anatomically related compartments as the potential source. Nevertheless, primary pathologies arising within this space should also be considered in the appropriate clinical context. Being familiar with the anatomical connections and both common and uncommon diseases of this area is essential for reaching the correct diagnosis. CT and MRI play a pivotal role in detecting diseases involving this area. Careful evaluation of this compartment should therefore be incorporated into routine abdominopelvic imaging evaluation to improve diagnostic accuracy and guide clinical management.

## Supplementary information

ELECTRONIC SUPPLEMENTARY MATERIAL

## Abbreviations

CT: Computed tomography
MRI: Magnetic resonance imaging
